# The Hospital Management and Treatment of Infectious Diseases.—I
*A paper read before the Royal Sanitary Institute, Leeds.


**Published:** 1922-09

**Authors:** A. E. Pearson

**Affiliations:** (Medical Superintendent, Seacroft Infectious Diseases Hospital)


### THE HOSPITAL MANAGEMENT AND TREATMENT OF INFECTIOUS DISEASES.*—I.

By A. E. PEARSON, L.R.C.P., M.R.C.S. (Medical Superintendent, Seacroft Infectious Diseases Hospital).

To review 32 years of hospital and fever work, comprising the care of 62,000 persons, irrespective of staff, and to compress it into a single paper, is a somewhat difficult task. In a hospital of the size of the one at Seacroft there are many and diverse problems, involving much responsibility and anxiety for the administrator. For, apart from purely medical, surgical and bacteriological work, the range of duties is extensive and various.

Of great concern are financial matters, as they are to the Committee and Council. The annual expenditure was large before the war. During and since the war, the cost on revenue account has become almost prohibitive. The cost is largely accentuated by the wide distances between the 61 buildings comprising the hospital, distances varying from 80 to 200 feet. (Dr. Thorne Thorne's allowable distance in his Memorandum was 40 feet.) These buildings, 61 of them, have to be reached by, and linked up with, hot-water pipes from the central system for hot water and heating, by gas and cold-water pipes, by electric cables and wires, and by corridors, subways, roads and drains. Repairs and replacements to this extremely wide system are considerable and costly, and necessitate, by the wide divergence of the constituent units of the hospital, the employment of a large staff of workers. My advice to those projecting hospital schemes is, for economical working, to lessen the distances adopted at Seacroft. That the size of the hospitals at Seacroft may be realised, I may say that there are :—

8 miles of drains.

26 miles of gas, water and steam pipes.

46½ miles of electric wires.

98,000 square feet of glass.

4½ miles of eaves spouting.

1¼ miles of cart roads.

1½ miles of covered corridors with subways 4 feet high.

Having in mind these details, the large cost entailed in working can, I think, be understood.

### On Accommodation.

There are 500 beds at the Seacroft Hospital, each having 2,000 cubic feet of space, of which there are 70 for the isolation of cases of doubt, and of mixed infection. In a general way the diseases dealt with are those scheduled in the Infectious Diseases (Notification) Act. Diseases not scheduled, as measles, chicken pox, whooping cough and others, are only taken to hospital when accommodation allows of their being taken with safety to themselves and the patients in the hospital. My view is that fever hospitals should not be overcrowded. An effort, I think, should be made to treat certain infectious cases at home, when the hospital has been filled to a certain extent. Especially, I think, home treatment should be carried out in the larger houses in which separate accommodation could be provided. This would allow of necessitous cases coming into hospital.

Fairly frequently cases are sent in with other infectious diseases running concurrrently. To prevent foreign or mixed infectious conditions spreading to others is a somewhat difficult matter, and one of great concern to the management. The incidence of these causes annoyance and distress to patients' friends, and tends to prolong the period of isolation. At Seacroft, as in other fever hospitals, the means of isolating these I consider insufficient, and, in my opinion, 50 per cent. of the available accommodation in a fever hospital should consist of single or two-bed isolation rooms, *e.g.*, large wards on the cubicle system. The "barrier" system in theory is excellent. In some hospitals the "barrier" arrangements are elaborate; but with a constantly changing staff the system may, I think, fail, owing to lack of knowledge or care in the nurse. Education of nurses in fever hospitals is most important; not only in aseptic methods of treatment, but in diagnosis and treatment, a careful, well-trained nurse is a great help. Much has been done in this direction by the Fever Nurses' Association.

There are 15 large pavilions at Seacroft, taking 30 patients each, these again being divided into two wards of 15. In these the patients are classified as to degree of sepsis, proceeding as they lose their acute and septic states to semi-acute and convalescent wards. This classification I regard as very important. This arrangement tends to prevent the supervention of added disturbances from contact with persons or things having more virulent or malignant micro-organisms, and with patients with concurrent infections. For, in my view, cases supposed to be of the same disease are not always alike from a bacteriological standpoint, though supposed to be.

### On Prophylaxis.

Prophylactic measures are most important. The members of the staff and the patients must be protected, so far as is possible, from disease. Much has been done with satisfactory results; much remains to be done. The time possibly may come when members of a fever hospital staff will be expected to possess, by vaccination, specific receptors, agglutinins or other antibodies for every known infectious disease. There appears to me to be one problem to be considered in connection with vaccines for prophylaxis, and that is the probable average length of time immunity lasts, which has not yet been defined, and the question of re-vaccination.

* A paper read before the Royal Sanitary Institute, Leeds.

In the case of typhoid fever it has been my practice for some years to vaccinate members of the staff engaged in attending to the typhoid sick against the disease, the result being that during this period there has not been one case of typhoid, though we rarely are quite without typhoid patients. Formerly and in the earlier years of my hospital work, there had been a fair number of attacks amongst the staff, with unfortunately fatal consequences in some. To protect satisfactorily and apparently perfectly against typhoid has been a great relief to my mind. In carrying out typhoid prophylaxis, I suggest as important that injections be continued if necessary beyond the usual two and till the individual's serum produces a fairly quick agglutination test.

Let me take another disease—influenza. At the hospital in the terrible pandemic of the year 1918 we lost 12 members of our staff of 80 attacked. No treatment, vaccine or otherwise, availed to mitigate its malignancy. During, and in the earlier part of, the present outbreak, which had been preceded by three serious cases in the staff, I have caused some 120 of the members of the hospital staff to be vaccinated with the Ministry of Health's anti-influenza vaccine. Of this number three only have developed influenzal symptoms, two of mild and one of moderate severity. All have recovered without secondary complications. This is in marked contrast to the incidence amongst the patients, in whom the attacks have been comparatively frequent, though of a somewhat mild kind. The incidence amongst our staff compares very favourably with that of other hospitals I know of, in which anti-influenza vaccine has not been used, and in which deaths from the disease have occurred. Some vaccines probably do not affect, to any great extent, the primary toxic disturbance, but they do, in my opinion, modify or prevent the secondary, often septicæmic, disturbances which are so much to be feared in infectious diseases generally. I can confidently commend the use of anti-influenza vaccine in all institutions. Incidentally I would ask the question, What are the causes of influenza and its rapid spread ? The cases I have come across in hospital do not seem to arise from personal contact, as they spring up in wards far apart. We must, I think, at least to some extent, look for meteorological conditions, which even now, to my mind, are not thoroughly understood in relation to the causation of infectious diseases.

Now let us consider what prophylactic measures can be adopted in diphtheria. The present means for prophylaxis, to some extent satisfactory, are not entirely so. For in spite of protecting gauze masks and antiseptic throat applications, nurses get diphtheria, and there have been several deaths. To prophylact attendants with antitoxin, small doses, say 500 units, might be used, but its influence is temporary and immunity soon passes away. Were these prophylactic injections continued, say every two months, unpleasant if not serious anaphylactic disturbances might arise. For some years it has been my practice temporarily to immunise scarlet fever and measles patients of susceptible ages against diphtheria, with more or less complete success, by giving small doses of antitoxic serum. I have done the same with success for orphanages, schools and o hers, in which diphtheria has occurred, the members of which have been sent up to the quarantine station for disinfection and observation. I have not found serum sickness or anaphylactic symptoms arise, except in very few cases, and in this way I have practically eliminated the occurrence of diphtheria in the scarlet fever wards. Before adopting this method diphtheria attacks would occasionally arise in the scarlet fever wards. A report on diphtheria was recently presented to the Ministry of Health by Dr. Copeman, in which prophylaxis is advocated by a toxin-antitoxin preparation in conjunction with the Schick test of susceptibility. I am pursuing this latter test at the present time in the diphtheria wards, but as yet have not had sufficient experience of it to form an opinion as to its positive value. I am about to test the value of prophylactic injections of toxin-antitoxin on those of the nursing staff who are willing to submit to the operation. I have no data on which to base an opinion of its immunising effect; it seems to me, however, that it may be of great value in producing a longer period of immunity than antitoxin alone. It is said to give a five years' immunity, but it seems that, as in vaccination against smallpox, reactionary disturbances may supervene. This may prove a troublesome factor in its application. In effect, the proposition would be to induce the production in the human body of specific receptors anticipatory to a diphtheria attack, rather than, as in antitoxic serum, to introduce receptors already made from an animal immunised exactly in the same way, with possibly better and longer immunity.

There is yet another infectious disease very prevalent and occupying much of the accommodation in fever hospitals. Can we provide defensive measures against scarlatinal attack ? The usual method, and the only one practised, so far as I know, is to endeavour to antisepticise the fauces by local antiseptic applications. I am inclined to believe that a mixed streptococcic vaccine which I now use for treatment might be used for prophylaxis purposes, much in the same way as a typhoid vaccine is used. I have no experience of its use in this direction.

*(To be concluded)*.

---

Automatically operated telephone exchanges have been on their trial in England during the last decade, and their success in eliminating operating errors and delays has been given a marked impetus to their general adoption. The exchange at Fleetwood, Lancs, is the first of its kind opened for public, as distinct from private, service. All modern telephone systems, whether manual or automatic, utilise small electro-magnetic " relays " to a very large extent for controlling the various electrical circuits ; but the relay system installed at Fleetwood performs the whole of the work of establishing and controlling subscribers' connections entirely by means of such " relays," without the intervention of mechanical switches or continuously rotating shafting. The whole of the service is automatic, the nearest operator being at Blackpool (a distance of 8 miles) ; even trunk calls, which are negotiated via Blackpool, are set up automatically at Fleetwood. The operator's salutation, " Number, please," is replaced by a humming tone, which is audible within a fraction of a second of lifting the receiver. The number is obtained by rotating a numbered dial, a signal indicating the ringing of the distant subscriber's bell. The connection is severed with the same speed. The Post Office has now 13 automatic exchanges in use, and others will be added.

